# La Clinique des Hôpitaux des Enfans, et Revue Retrospective, Medico-Chirurgicale et Hygienique

**Published:** 1842-07-01

**Authors:** 


### LA CLINIQUE DES HOPITAUX DES ENFANS, ET REVUE RETROSPECTIVE, MEDICO-CHIRURGICALE ET HYGIENIQE. Paris, 1841—2.

### The Clinical Practice of the Hospitals for Children, and Retrospective Review, Medico-Chirurgical and Hygienic.

THE object of this new periodical, which is now being published under the auspices of several eminent physicians and surgeons in Paris, as Baudelocque, Jadelot, Guersant, and others, men who have devoted and still devote their exclusive attention to the study of the diseases of infancy, is to present, 1st, the medico-chirurgical clinique of the hospitals destined for children;—2nd, the retrospective review of all that has been written on the diseases of children;—3rd, the therapeutical management of children;—4th, the hygienic review, embracing all the questions of hygiene and of preventive or prophylactic medicine, considered in their relations to infancy. The prominent feature of this journal, according to the editor, is to consider questions of a decidedly practical nature, and such facts as are of every-day occurrence.

He accordingly proposes to explain the character of the work in the following condensed formula: "the clinical history of those facts which are most frequently met in practice, observed more especially with reference to diagnosis and therapeutics." Exceptional or extraordinary facts, however, which occasionally occur in practice, are not to be rejected from the pages of this journal; still they are not to be sought out with the view of making them the principal point of attraction. From the conviction that nothing is more adverse to the progress of science, than the desire of the marvellous, the editor of the Clinique des Enfans repudiates the practice of presenting to his reader's attention nothing but extraordinary exceptions and novelties, feeling that he will render his journal more useful by seizing those delicate shades so frequently met in the ordinary facts of infantile medicine, and which render its practice so very difficult. The importance of studying infantile disease, as a separate branch of medical study, is obvious to every one, who will take the trouble of considering the peculiarities of infantile life.

Every one engaged in the practice of medicine soon finds out the difficulty of arriving at a correct diagnosis in examining children in early life. The organs of relation being then but very imperfectly developed, the medical attendant is in such cases entirely destitute of that valuable information, which he might readily obtain from an adult patient. Another source of difficulty is, that in consequence of the excessive sensibility of the infant, the sympathetic phenomena are very marked, and oftentimes become confounded with the idiopathic symptoms of the disease. We must admit our continental neighbours have the advantage of us, in the numerous facilities they enjoy for studying the diseases of children. Their Hôpital des Enfans Malades has nothing at all approaching it in these countries. Such establishments are absolutely and essentially necessary for attaining a knowledge of infantile pathology.

According to the plan of the Clinique des Enfans, as given in the introductory chapter, each number is to contain interesting cases of the principal diseases to which children are liable, with practical remarks on them, and also general treatises by the most distinguished professors engaged in the department of infantile medicine on the pathology, therapeutics and hygiene of infants. We shall present our readers with a condensed analysis of the most practical articles of this journal, and in so doing we shall endeavour to adhere to the order in which the several numbers have been published, as far as may be consistent with convenience. A strict adherence to the precise order, however, observed in the original journal, would in many instances occasion a want of connexion, and thereby involve in some obscurity, articles in other respects valuable. We shall commence by giving an analysis of an interesting case of croup, in which it was deemed necessary to have recourse to the operation of laryngo-tracheotomy.

### Clinique Medicale.

### *Case of Laryngo-Tracheotomy for Croup, in the Sick Children's Hospital.*

By M. A. Contour.

Tracheotomy, as a means of treatment in croup, is too well established in medical science by its great success, to have any thing to apprehend from the publication of some few unsuccessful results; many of which might be attributed to the operation having been too long delayed, and not undertaken until the pathological changes had proceeded too far.

The 18th of February, 1841, a boy, seven years old, was taken into the hospital, under M. Bonneau.

The child appeared to be of rather a good constitution. He complained merely of slight headache, and some colicky pains, and of diarrhœa—no fever—respiration free and easy. On the following day he complained of considerable soreness of the throat—could breathe only through the mouth, the nose being stopped with thick mucus.

On examining the posterior fauces, redness and swelling of the isthmus faucium was observed, as also a false membrane on the right side of the velum palati, and another on the tonsil of the same side—no swelling of the sub-maxillary glands—the child answers questions, but his voice is slightly changed. Cough hoarse, and already evincing somewhat of the croupal character. Breathing not embarrassed—percussion elicits a clear sound in every part of the chest—the vesicular expansion audible over every part.

(Tartar emetic in warm water—aluminous insufflations—hot cataplasms to the feet.)

20th. The tartar emetic made him vomit several times, and in the matter vomited a false membrane seven or eight lines in length, with four or five in breadth was found; it was thick and hard, and was supposed to be that seen the day before on the right side of the velum; other fragments of false membrane were also found, some white, and others greyish. The child was relieved after discharging these false membranes. The alum insufflations were continued, and the rest of the day passed off very well, but he spent a bad night, and became delirious.

In the morning a remarkable change was observed to have taken place since yesterday—countenance presented a peculiar aspect—it was dejected and pale, with a purple tint on the cheeks—eyes dull, and the upper eyelid evinced a constant tendency to cover the globe of the eye. The pupil, without being either dilated or contracted, retained its natural sensibility. Breathing very difficult, without, however, that laryngo-tracheal whistle so often observed in croup being heard; at each inspiration the alæ nasi became very much dilated and the shoulders raised. The lower lip was depressed at each inspiratory movement, and the commissures of the lips were drawn slightly outwards—thirty-two inspirations per minute. The patient, whose intellectual faculties are for the time intact, has still sufficient strength to sit up, and on examining his throat, the false membrane observed the day before on the side of the velum is no longer visible; the place it occupied bleeds a little. The rest of the velum and the uvula are covered with a whitish false membrane of recent formation, and which seems to be very thin. On introducing a spoon into the bottom of the throat, to examine the isthmus faucium, we brought up thick, whitish mucus, mixed with streaks of blood.

Auscultation of the larynx detects a peculiar and very strong sound, which seems to be produced, either by a thick, moveable mucus, or by a floating false membrane, agitated by the air at its entrance into and egress from the air-passages. The respiratory murmur in the chest has no longer the natural softness of the vesicular expansion; it is rough, and, as it were, ill-formed, and the reverberation of the sound formed in the larynx is heard on both sides. The voice is extinct, and the patient can answer only by signs, or in a very low voice. The cough is decidedly croupal. At each fit of coughing the respiration is whistling. The diarrhœa was rather increased by the tartar emetic—heat of skin natural;—pulse 160, weak and compressible.

(Syrup of ipecacuan—two small blisters along the course of the carotids—insufflation of alum in powder; hot cataplasms to the feet, &c.)

Sometime after the visit delirium came on, and the breathing became more and more embarrassed; towards noon the little patient lost all consciousness; the face, which was covered with sweat, presented a bluer tinge than in the morning; lips purple—respiration very laborious; tracheal râle heard at some distance; asphyxia seemed to be approaching, and the child appeared on the point of dying; pulse 172. Some strength still remained. Tracheotomy was now determined on, and resorted to.

Though the operation lasted but a few minutes, still towards its termination the patient was attacked with syncope, which lasted for about ten minutes. In about half an hour after the pulse was 156, and the respiration 32. An attempt was made to raise the patient's strength by administering some vinous sugared water, but he could not swallow, and the liquid fell into the air-passages. Sinapisms were applied to the thighs, and in about an hour after he was able to swallow a few spoonsful of the sugared wine-water. By the contraction of the muscles of the face, it was evident he felt the pain of the sinapisms. He was now perfectly come to himself, and seemed to have recovered strength; he was able to place himself on his right side, to support his head with his hand, to put out his tongue, and to answer some questions put to him by making signs; the face, still pale, was less blue—pulse now 144; the respiration was now regular and easy; in a word, he was much better than before the operation. This amendment, however, was but of short duration: delirium came on again, and the child made several attempts to leave his bed; the respiration became embarrassed, and the symptoms of asphyxia re-appeared. Matters became worse and worse, and the child at length died seven hours after the operation.

*Autopsy*, thirty-six hours after death. Temperature rather cold; a little moisture.

*External Appearance.*— Body well formed—no rigidity.

*Cranium.*—Cerebral membranes much injected—veins perceptibly distended with black blood. The adherence of the membranes to the brain not abnormal. Cerebral substance of a good consistence—no serum in the ventricles.

*Air-passages.*—Posterior fauces lined by a false membrane which is observable equally on the entire anterior surface of the velum palati, on the uvula and the tonsils. But it does not every where present the same characters; on the velum it is of a yellowish, white colour, thick and elastic, and easily separated from the mucous membrane, to which it is attached by small filaments. On the uvula, on the contrary, as also on the tonsils, it is of a grey colour, is much less thick and consistent, and it breaks when an attempt is made to remove it; the subjacent mucous membrane is slightly injected. On the posterior surface of the velum this false membrane presented the same differences as on the anterior—the false membrane of the body of the velum was more dense and resisting than that of the tonsils and uvula, where it is somewhat soft, and resembles a sort of detritus of a dirty grey colour. This substance covers all the internal surface of the pharynx, as far as the thyroid cartilage, sometimes in the form of well-formed false membranes, varying in size and thickness, sometimes in the form of that species of greyish detritus easily raised with the finger, whilst the truly membranous parts adhere more closely to the subjacent mucous membrane, which is every where injected and presents a bright colour.

The false membrane on the posterior surface of the velum palati ascends into the right nasal fossa, of which it lines the inner surface formed by one of the sides of the septum; but it has this peculiar character, that being thick inferiorly, it diminishes perceptibly according as it ascends, and is very thin in the upper part.

The epiglottis on its anterior and posterior surface, and the larynx through its internal surface, are lined with false membranes of less consistence and thickness than those of the velum palati, but they adhere closely to the mucous membrane. The cordæ vocales are entirely covered, and on viewing the larynx when opened posteriorly, we can neither see the ventricles, nor the projection of the thyro-arytenoid ligaments.

The trachea through its entire length, from the first cartilaginous ring to the bifurcation, is red and very much injected, and there are observed here and there some traces of false membranes. This, however, may be accounted for by the fact of our having had recourse several times, after the operation of tracheotomy, to cleansing out the trachea. The proof, that this was the means of destroying the false membrane, is, that this membrane is found at the bifurcation of the trachea, and it may be traced in the bronchi of the left side as far as the fourth ramifications: no trace can be discovered in the bronchi going to the right lung; these present merely a very marked redness and injection without the least tumefaction. The false membrane of the left bronchi, of a dull white colour and extremely thin, adheres but slightly to the subjacent mucous membrane; according as we advance into the bronchial ramifications, it becomes less formed, and terminates in a number of small white points, deposited in a light transparent tissue.

The two lungs present numerous traces of inflammation. The left lung, more involved than the right, is hepatized over the entire lower half of the upper lobe. In the lower lobe some points of hepatization are observable. The right lung, which is engorged posteriorly, presents in its three lobes a considerable number of circumscribed points of lobular pneumonia in the stage of red hepatization. No trace of pulmonary tubercles. The bronchial glands and the pleura healthy. Heart of the normal size, and its parietes of a natural consistence—no serum in the pericardium—stomach in the normal state.

*Small Intestine.*—Through the entire extent of this intestine the glandulæ solitariæ present the appearance of small globular bodies of a dull white colour, but they are neither softened nor ulcerated. Peyer's glands are considerably developed, and present a peculiar appearance; they are all, or nearly all of a violet colour, and the mucous membrane covering them, without any alteration either in thickness or consistence, presents sometimes mamillated elevations, some of them with a broad base, others with pedicles, resembling in some degree small mucous polypi; sometimes it presents folds of greater or less regularity which may be compared to the valvulæ conniventes—nothing remarkable in the liver, spleen, kidneys or bladder.

On perusing this case we shall find that croup does not always faithfully follow the course assigned to it in books.

Thus the hoarse cough never even for once came on in kinks; it occurred from time to time, and presented itself under the form of one or two paroxysms followed by a whistling inspiration. The laryngo-tracheal sifflement usually heard at a distance, at each inspiration, did not exist for an instant in the case before us. Delirium and convulsions, two symptoms so rarely observed in such cases, occurred in this case.

This case is also a good instance of those croups in which it is impossible to meet the three stages described by authors, and the course of which is so rapid that the medical treatment has not had time to act. Thus we see the disease commence on the night of the 18th—and on the noon of the 20th, that is, about thirty-two hours after its commencement, the physician was obliged to have recourse to a surgical operation. Probably in those cases where the course is so rapid, valuable time should not be lost in employing means, which, if not always, nearly always, are found powerless, but we should have recourse at once to operation, before the pseudo-membrane has seized on the trachea and bronchi. Indeed this case comes in support of this opinion—accordingly we see that the operation, though practised a few hours after the commencement of the disease, was resorted to at too late a period; for, as the autopsy proved, the false membranes had already extended into the first ramifications of the left bronchi, the pulmonary tissue was already attacked; circumstances which diminished considerably the chances of success.

Case.—*Bright's Disease after Scarlatina: Anasarca; Albuminous Urine; Œdema of the Lungs.*

On the 16th of February, a little girl, four years of age, born of healthy parents, entered the hospital: she had been vaccinated, and was in the habitual enjoyment of very good health, when about a month previously, after several attacks of vomiting accompanied with fever, she was seized on the same day with intense redness of the anterior part of the chest, as also of the back; this was all considered as the result of indigestion, and at the end of a few days she left the hospital. Eight days after some degree of swelling was observed; it commenced in the right hand, extended to the left, to the lower extremities, and then to the face. The mother stated that the child had fever every night, that her urine was very dark, and that she had a little purging. Present state on the 16th.—The child had a very fair skin, was very intelligent, and appeared to be of a strong constitution; face not œdematous; eye-lids not puffed; cheeks rosy; upper and lower extremities tumefied, tense, elastic, do not retain the pressure of the finger; abdomen not tense, nor does it appear to contain any liquid; the respiratory murmur perfectly pure all over the chest; nothing abnormal elicited by percussion; pulse 80 and regular, not full; heat of skin moderate; appetite good; ordinary thirst. She is very lively, and has the appearance of being in good health. There was very little change in her up to the 24th, her urine still continuing of a deep yellow colour, and nitric acid and heat throwing down a whitish flocculent precipitate. The vapour bath was employed twice, and seemed to have the effect of rendering the urine less albuminous, and of diminishing the tense darkness of the limbs.

24th. During the preceding night she was restless; in the morning her face was a little swollen, and the limbs were increased in size, without, however, retaining the impression of the finger; skin hot; pulse increased in frequency—urine the same as on the preceding days—appetite diminished—during the night she was restless, and towards morning had some bilious vomiting.

25th. At seven in the morning she was seized with intense dyspnœa—œdema perceptibly increased—eyelids œdematous, and diminish the aperture of the eyes—the left side of the body, on which the patient prefers to lie, more infiltrated than the right; pulse small, (160 per minute); breathing very short and rapid (84 per minute); alæ nasi widely dilated at each inspiration; pulsations of the heart very rapid, its impulse of considerable strength, the sounds very distinct. A little sub-crepitous râle is heard posteriorly, at the base of the left side of the chest. An attempt was made to bleed her from each arm without success, owing to the great infiltration. She vomited some aperient medicine given to her; and in consequence of the intense dyspnœa, and the increasing œdema, six leeches were applied to the precordial region, but without any improvement. All the symptoms becoming more aggravated, she died on the 26th at six o'clock in the morning.

*Autopsy*, thirty hours after death.—Œdema more marked on the left than the right; the back of the left hand, though much infiltrated, scarcely retains the impression of the finger.

*Brain*.—Some drops of transparent serum in the lateral ventricles.

*Abdomen*.—Liver large, of a very deep brown red; its vessels are gorged with blood.

*Kidneys*.—The left kidney about eight centimetres in length and four and a half in breadth; pale and somewhat yellowish; it presents a small number of whitish granulations externally. When its convex edge is cut into, its mamillæ are observed to form a contrast by their bright red colour, with the pale cortical substance; the latter, more especially in the portions separating each mamilla, presents a considerable number of small whitish points about the size of a pin's head; these granulations are irregular, not prominent, and do not appear distinctly separated from the cortical substance. The right kidney, eight centimetres in length and four and a half in breadth, is not so pale as the left, but yet is far from having the normal colour. Its tubular substance, when it is cut into, is redder than that of the organ of the opposite side; the same may be said of the cortical substance interposed between each cone.

*Thoracic Cavity*.—No pleuritic adhesions; nearly a glass of transparent citron-coloured serum effused into the left pleural cavity; only about half that quantity into the right. A small quantity of serum found in the pericardium.

*Lungs.*—The left very heavy: with the exception of the apex of the upper and lower lobes, the lobules of which are rose-coloured, all this lung presents the colour of wine-lees; it no longer crepitates, nor does it present the natural vesicular structure; some portions of this lung, when put into water, sink to the bottom; they are hard, and easily torn, without, however, presenting that loss of cohesion peculiar to pneumonic hepatization; when this pulmonary tissue, so changed, is torn, there flows from it a considerable portion of serum, somewhat frothy, reddish, and inodorous. The right lung is much less heavy than the left. No tubercle was found in the lung. The bronchial glands natural.

*Remarks.*—The anasarca of infancy most frequently supervenes on some of the exanthems, as scarlatina; the fever, which sometimes is accompanied by slight redness of the skin, may pass off without being noticed by the child's friends; when they are interrogated on the subject, as to whether the child has had scarlatina before the anasarca, they answer in the negative; but if the physician, not content with this reply, questions them regarding the illness of the child a little before the appearance of the dropsy, he often arrives at the knowledge of the cause, which otherwise would have escaped him. We have an instance of this in the case just given; for the child's friends took the commencement of the scarlatina for indigestion. The eruption, to be sure, was slight; but it is well known that there is no relation between the intensity of the eruptive fever and the frequency of the dropsy.

From the existence of the scarlatina having been overlooked, the child left the hospital after a few days; after this she was exposed to cold, a powerful, though not an indispensable cause of dropsy, as we sometimes see patients attacked with anasarca before they have left their bed. When this child entered the hospital, the anasarca was of three weeks' standing; the sub-cutaneous cellular tissue, though infiltrated, was resisting and elastic; skin did not present that bloodless colour so frequently accompanying dropsy; the urine was constantly albuminous. In this state of things, we were warranted in supposing that the albuminuria depended on Bright's disease. Still so rapid a death was not anticipated.

---

We find in the 2nd Number, p. 66, a very interesting Review of a Thesis of Dr. Baron, an intern of the Hôpital des Enfans Malades, of which we shall now give an analysis. The entire of the review of this Thesis extends through the 2nd, 3rd, and fourth numbers of the Journal. The subject of the Thesis is—

*Pleurisy in Children.*—M. Baron considers this affection as it occurs in children from birth to their fifteenth year. Out of 403 autopsies, the author detected traces of pleurisy 159 times, that is, in more than one-third of the entire.

We shall commence our analysis by as rapid a sketch as possible of the ætiology of this affection, as we well know that a knowledge of the causes of disease may become, especially in doubtful cases, a powerful element of diagnosis, and very frequently a source of valuable indications.

*Physiological Causes.*—1st. Influence of *age.*—Billard tells us that the fœtus is not exempt from this disease.

From considerable experience, M. Baron concludes that pleurisy is much more frequent in children from two to fifteen years, than in the first two years of life. In 3,392 autopsies of children from one to two years old, he found 205 indisputable pleurisies, and 79 pleuritic changes which were probably not inflammatory. In 181 autopsies of children from twelve to fifteen years of age, he found 158 pleurisies and 13 pleuritic alterations. With respect to the comparative frequency of pleurisy at the different epochs comprised between birth and the fifteenth year, it sets in more frequently during the first five days, than during the period included between the fifth day and the first month ; it diminishes in frequency after the first month up to the second year; from the second to the third year it is much more frequent than from the third to the fourth, and more especially from the fourth to the fifth. It is still more rare during the following years, chiefly between the thirteenth and fourteenth year.

2nd. Influence of *sex.*—It is not till the approaches of puberty, that we commence to appreciate, in most diseases, any modifications depending on sex. With respect to pleurisy, M. Baron has observed it to be somewhat more frequent in males.

3rd. Influence of *constitution* and of *habitual health.*—The greater number of children observed by M. Baron were of a slender and delicate frame; with debility either acquired or hereditary; small size, small limbs, bad diet, want of due care, &c. In some a degree of marasmus more or less advanced was observed. Rickety malformation of the chest does not seem to exercise much influence, whilst the position in which children are constantly placed may have some influence on the development of pleurisy.

*Pathological Causes.*—Influence of *previous diseases.* The diseases which may become complicated with pleurisy are the following:—1st, *Pleurisy* itself is occasionally observed to return in the form of a relapse, more frequently in subjects in the second infancy, especially in tubercular subjects, than in those of the first; but these relapses, in M. Baron's opinion, seemed to depend rather on the general state, on the constitution of the subject, or on the state of the other organs, more especially of the lung, than on previous pleurisies.—2nd, Passive pleurisy, which must be admitted in the case of children as well as passive pneumonia, a congestion which manifests itself by the physical signs of pleuritic effusion, without symptoms of inflammatory re-action, may become the cause of pleurisy. 3rd, The influence of *pneumonia* on the formation of pleurisy is marked by the frequent coincidence of inflammation of the pleura with that of the lung. It is in new-born children we most frequently meet pleurisy without pneumonia, and it is in those of from a month to a year that this simple pleurisy is most rare. M. Baron has met pleurisy as often in children of from two to six years as in those of from six to fifteen. 4th, Pneumonia terminating in vomica, or abscess of the lung, seems to have occasioned the development of pleurisy in some cases. 5th, *Gangrene of the lung*, and 6th, *Pulmonary apoplexy*, have been occasionally found to coincide with pleurisy. 7th, *Infiltration of the lung* often coincides with simple serous, or sero-purulent effusion. Those pleurisies which coincide with an acute œdema of the lungs which has come on during Bright's disease, are rather the effect of a common cause, than the effect, the one of the other. 8th, Pleurisy may precede *bronchitis;* the contrary, however, happens more frequently; in such cases it generally, though not always, happens, that pneumonia is the medium of transmission between bronchitis and pleurisy. 9th, *Croup* is said to be frequently complicated with pleuro-pneumonia. 10th, *Simple laryngitis*, or *stridulous laryngitis* are sometimes complicated with pleurisy. 11th, When *hooping-cough* is complicated with pleurisy, there generally co-exists at the same time a pneumonia. 12th, *Millar's asthma*, in the *acute* form, may be complicated, if not with pleurisy, at least with something very like it. 13th, During *chronic asthma* (pulmonary emphysema) pleurisy may supervene in children. 14th, *Coryza* may be accompanied with pleurisy in very young children. 15th, *Pulmonary tubercles* constitute the most frequent pathological cause of pleurisy; instances are constantly observed of this. We mean here tubercles properly so called, especially when they are softened. We find general adhesions more frequently with cavities than with tubercles still in the crude state. With respect to granulations they frequently exist without the slightest lesion of the pleura. *Tubercular pleurisy* is very rare before the first year. It is more rare from the seventh to the eighth, from the ninth to the eleventh, and from the fourteenth to the fifteenth. 16th, The influence of *tubercles in the bronchial glands* and in the mediastina on the production of pleurisy is rather doubtful. 17th, Abscesses in the mediastina, whether tuberculous or not, have been found by the author the manifest cause of the development of pleurisy. 18th, *Pericarditis* and carditis may follow pleurisy, or the reverse. 19th, *Inflammation of the thoracic parietes.* Pleurisy has been found to follow swelling of the skin, cellular tissue and muscles of the parietes of the chest, such inflammation being occasioned by a leech-bite. 21st, *Gangrene of the mouth.* Frequent examples are recorded of this complication. 22nd, *Gangrene of the pharynx, and œsophagus* may be complicated with pleurisy. 23rd, *Aphthæ* may be complicated with pleurisy. 24th, Pleurisy is sometimes attributed to the presence of *worms* in the alimentary canal. 25th, Diseases of the liver, as hepatitis, congestion of this organ, cirrhosis, &c. have been observed to be accompanied by pleurisy; 26th, *Jaundice* sometimes is accompanied by pleurisy; whilst 27th, *peritonitis* frequently coincides with pleuritis in infancy. 28th, *Bright's disease*, in almost all the children affected with it, is accompanied by some morbid change in the pleura, either simple effusion, or pleurisy. 29th, Certain *encephalic affections* (meningitis, hydrocephalus) are accompanied by pleurisy. 30th, *Anasarcous depositions*, essential œdema with or without albuminuria, are combined with pleurisy: thus it is found in œdematous new-born children. These two diseases are probably effects of one and the same cause. 31st, The same may be said of *induration of the cellular tissue*. 32nd, *Gan-*

*grenous affections* in all their forms, appear to exercise some influence on the development of pleurisy. 33rd, *Diseases of the skin,* more especially the acute exanthems, are frequently complicated with pleurisy; *measles* more frequently than any other. 34th, *Chronic cutaneous affections,* so frequent in children, are considered as the causes of pleurisy. 35th, *Scrofulous affections* predispose to pleurisy, whether from the presence of tubercles in the lungs or in the mediastina, or from the delicacy of the constitution. 38th, Surgical diseases, great operations and phlebitis, may be followed by pleuritis.

*External Influences.*—1st, *Tedious labour,* especially when the child remains a long time in the passage, may become a cause of phthisis, this condition favouring the sanguineous exhalations into the pleura. 2nd, *Hereditary predisposition* may have some influence. 3rd, *Lactation,* when imperfect, and received from a woman who is unhealthy, or pregnant, may, by weakening the constitution, predispose to pleurisy. 4th, *Cold* and sudden alternations of temperature, have an unquestionable influence on the production of pleurisy. There can be no doubt that the surrounding cold air is one of the principal causes of pleurisy in new-born children. 5th, The influence of *seasons* corresponds with the preceding. In Winter pleurisies are most frequent; a little less so in Spring and Autumn. 6th, The influence of *contusions,* those more especially on the chest and accompanied with compression, has been proved by numerous examples.

*Symptoms.*—*Local Symptoms.*—1st. *Pain.* In very young children the means of ascertaining the existence of this symptom, is to percuss the chest; this will be sure to cause pain and so discover the precise seat of the disease. Great caution and attention are required on this point, especially in new-born children. This pain, thus excited, may be recognized by a mere grimace, or by a shrinking back on the part of the child. The seat of the pain varies; sometimes it is behind the sternum, sometimes laterally, either at the middle, or at the lower part of the chest; sometimes on a level with or below the false ribs, in the epigastrium, at the shoulders, behind the scapula of the affected side, and lastly in the back.—2nd. *Dyspnœa* is a symptom detected in the child by the respiration being short and high, being accompanied by elevation of the chest, or considerable enlargement of the anterior abdominal parietes; there is also swelling of the veins of the neck, bluish tint of the lips, alternate dilatation and contraction of the alæ nasi, an expression of dejection in the countenance, a look of distress, prominence of the eye-balls, considerable separation of the eye-lids, a convulsive grasping at objects which may serve as means of support. These symptoms are sometimes intermittent and periodical.—3rd. *The Respiration,* ordinarily frequent, varies from 30 to 100 per minute, according to the child's age. Each respiration, which is very short, is separated from the others by intervals proportionally long. Seldom there exists merely increased elevation of the chest; more frequently the movement of enlargement of the lateral parietes is supplied by that of the diaphragm; thence, in pleuritic patients, there is an increase of the abdominal respiration. M. Baron says he has frequently seen the inferior ribs kept immoveable during a strongly abdominal contraction, whilst the other ribs performed their ordinary movement. 4th. The *Cough* is usually frequent and dry ; in the second infancy there are sometimes mucous or salivary sputa. 5th. The *Dilatation of the chest* by the action of the liquid effused has not exceeded a centimetre and a half. It is much more frequent on the right than on the left. Contraction of the cavity of the thorax is more frequent in tubercular pleurisies than in simple pleurisies. This deformation sometimes succeeds pleurisies not of a tubercular character. 6th. *Percussion of the chest.* The sonorousness of the chest may not be altered, or be so only over a small space, because the quantity of liquid effused is frequently very small. When the effusion is very copious, the dulness most frequently occupies the posterior and inferior part of the thorax. Sometimes it is limited to the posterior and upper part. It may be circumscribed at the anterior or lateral part, either superiorly, or inferiorly, or entirely from above downwards. It ordinarily extends from behind forwards, and from below upwards, then diminishes in the inverse order. Like pleurisy, it is more frequent on the right side. Often there is dulness posteriorly, and only diminished sonorousness anteriorly. The sonorousness, instead of being diminished, will be increased, if there be pneumo-thorax. In order to be able to diagnosticate morbid dulness and the tympanitic sound, it is necessary to know the relative sonorousness of the different points of the chest: in children of from eighteen months to seven or eight years old, the general sonorousness of the chest is very considerable. It is a little less in new-born children, than in children who have reached the first or second year. Anteriorly it is less than posteriorly; more especially less in the precordial region. In new-born children the limits of the dulness are difficult to be assigned, because the heart is almost always covered by the anterior part of the left lung. It is as far as the seventh rib nearly, from above downwards, that the sonorousness extends. On the right inferiorly, over a space of from four to six centimetres, the sonorousness is somewhat less than over the rest of the anterior region, by reason of the liver, which, being always large in children, projects more or less into the chest. On the left, the limits of the normal sonorousness are less fixed than on the right, in consequence of the frequent variations in the size of the stomach and intestines, occasioned by the frequent repetition of digestion in children, and the usual dilatation of the intestines by gases.

7th. *Auscultation.*—The effusion may be very small, or even none ; and then there may be no diminution of the respiratory murmur. There is an *absence of the respiratory murmur* only during the existence of the effusion. Besides, the respiratory murmur being very much developed, a considerable obstacle is required to destroy it. The presence of false membranes is sufficient to diminish the respiratory murmur, and at the same time the sonorousness. The *bronchial respiration* may be produced by effusion, but it is much less frequent than the absence of the respiratory murmur. The *dry and rough respiratory murmur* is frequently found. The *prolonged expiratory murmur* is often heard, which is explained by the compression of the lung. In children the expiratory murmur is prolonged by the slightest cause.

8th. The *resonance of the voice* is not oftentimes increased. In pleuro-pneumonia, on the contrary, it is increased by the pneumonia. When the effusion is considerable, there is oftentimes bronchophony and œgophony. *Bronchophony* co-exists with the *bronchial respiration. Œgophony* is rare in very young children ; it becomes more frequent according as children approach puberty.

9th. *Application of the Hand.* The tremor of the voice, felt by the hand applied to the parietes of the chest, disappears in pleuritis, in the part on a level with the effusion. The hand also detects the pleuritic friction.

*General Symptoms.*—1st. *Attitude.* The patient lies ordinarily on the back, often on the affected side, seldom on the healthy side. Sometimes however, lying on the affected side increases the pain, cough and dyspnœa, and becomes impossible. Often, at the commencement of the disease, the patient lies on the side; at a more advanced period it becomes indifferent. Sometimes, as long as the pain exists, lying on the affected side increases the pain; when it has disappeared, the little patient cannot lie on the healthy side, because it increases the dyspnœa. The dyspnœa is sometimes so urgent that the sitting position is the only one endurable. There is often a frequent change of position, and constant restlessness, especially in early infancy; occasionally complete immobility, or only a little languor, and often debility.—2nd. *State of the face.* Face pale; sometimes red at the onset, either on both sides, or only on one; occasionally livid. Physiognomy occasionally sad, and restless; sometimes expressive of pain and dejection; eyes sometimes dull at an advanced period.—3rd. *External appearance.*—Skin of the entire body pale, sometimes dry.—4th. *Jaundice.* Pleurisy is accompanied by this colour in new-born children. This, however, cannot be set down as a symptom.—5th. There is sometimes an abnormal *eruption of papulæ* during the course of pleuritis.—6th. *Emaciation* may proceed even to marasmus, when the pleurisy is of long continuance. —7th. *Œdema* sometimes occurs as a symptom of pleurisy. It is observed at all periods of infancy, especially in very young children, and it may be general or partial.—8th. *Induration of the cellular tissue* has sometimes been found to be the effect of pleuritis.—9th. *Fever.* In many cases there is intense heat at intervals, chiefly at the onset of the disease. When it tends to a fatal termination, there is a cold feel, either of the entire skin, or merely of the extremities. In inflammation of the chest in children the mammary region is hotter than the other parts. *Pulse* variable, more often frequent; when pneumonia co-exists, it becomes so frequent, that it is impossible to count it. Pulse is slow and irregular in cases, where there was cerebral complication. The fever is more frequently continued in acute pleurisy; in many cases there are exacerbations, most frequently towards evening, sometimes at night, and even during the day.—10th. *Palpitations* sometimes observed.—11th. *Cerebral symptoms.* Convulsions have been observed more especially in young children, head-ache, delirium, coma, dilatation of the pupils, strabismus, a contracted and rigid state of the limbs.—12th. *Abdominal symptoms,* especially in very young children; vomiting of drink and food, of bile, mucus, bloody substances, at the same time that there is sanguineous effusion into the pleura; dryness, redness, or mucous coating of the tongue; thirst, diarrhœa of yellow or green matter, tension of the abdominal parietes; loss of appetite; refusal of the breast.

*Course, duration, and mode of termination.*—Onset often abrupt. Passive pleuritis is developed slowly. Its commencement often difficult to be detected. The author lays down a list of the symptoms in the order of their succession; pain, great inquietude, fever, hurried respiration, slight dyspnœa; then, dulness, commencing usually at the posterior and lower part, and extending thence upwards and forwards; absence of the respiratory murmur, and in some cases œgophony, cessation of pain, crying, respiration more frequent; dyspnœa; cessation or diminution of fever, bronchial respiration soon replaced by the absence of the respiratory murmur; then a diminution of dulness, appearance of the vesicular murmur in points where it had ceased to be heard. If the disease be long continued, there are alternate remissions and exacerbations. Should the disease go on to effusion, the liquid makes an exit for itself, either by the air-passages, by means of a communication established between the cavity of the bronchi and that of the pleura, or through the thoracic parietes. There is an accompanying affection, which does not abridge the duration of pleurisy, and that is pulmonary phthisis—the same may be said of a scrofulous habit of body.

*Differential Diagnosis.*—There are two circumstances which may render the diagnosis of disease difficult, either the presence of certain symptoms which divert the practitioner's attention from some certain affection which does exist, or the absence of certain characteristic signs of an affection which really does exist notwithstanding the absence of these signs. In the latter class the author places certain circumstances whose absence may render the diagnosis of pleurisy difficult. Thus the small extent, as well as the small quantity of the effects of inflammation, the partial existence of pleurisy, the very small dimensions of false membranes, the trifling quantity of the effusion, circumstances which render the perception of the physical signs very obscure; want of the rough friction-sound of the false membranes occasioned by their slight consistence, short duration of the dulness on percussion, absence of the vesicular murmur, absence of bronchial respiration, infrequency of œgophony, and the uncertainty with respect to pain. In the first order we are to place the frequent coincidence of the different diseases of the air-passages or of the other structures more striking than the pleuritis, and attracting all the attention of the practitioner; thus, pneumonia, in consequence of its almost constant coincidence and predominance, and the analogy of its symptoms with those of pleurisy; pericarditis, by the similarity of the symptoms and the vicinity of the organs affected; congestion or inflammation of the encephalon, by the nervous phenomena accompanying them, especially convulsions, and which so often serve to mask the thoracic affection.

Convulsions occasion no real difficulty in the diagnosis of pleuritis in children, when we have examined the different organs, the chest in particular, except in those cases where the products of the inflammation of the pleura, especially the effusion, do not manifest themselves by sufficiently marked physical signs. The difficulty of detecting pleuritis is greater, the younger the child is. The principal affections which may be confounded with pleuritis are—acute and chronic pneumonia, infiltration of the lung, gangrene of the lung, pulmonary tubercles, bronchitis and croup; the acute asthma of infants, chronic asthma, pleurodynia, which latter is readily confounded with pleurisy, especially in children below three years old; pericarditis, with or without effusion; aneurysm of the heart and vessels; diaphragmatic hernia, &c.

It is with *pneumonia* that pleuritis is most frequently confounded. In pneumonia the red colouring of the cheek-prominences, dryness and incrustation of the lips, are more frequent than in pleuritis; acceleration of the pulse more constant, more continued; alteration of the face, depression of the strength more rapid; infiltration of the external cellular tissue is a sign of pleuritis, not of pneumonia. Hurried respiration more continued in pneumonia. Respiration rather abdominal in pleuritis, and costal in pneumonia; cough more sonorous, and less short in pleuritis than in pneumonia; cry more frequently veiled in the latter. Dulness more complete in case of effusion; still in new-born infants the dulness is sometimes very marked in pneumonia, and sometimes none at all in pleuritis. Crepitous and subcrepitous râle in pneumonia; but oftentimes accompanying affections of the lungs and bronchi give rise to râles during the existence of pleuritis. Bronchial respiration more frequent in pneumonia; absence of respiration more frequent in pleuritis. The very feeble respiration occurs more frequently in pneumonia, especially in new-born infants, and more rarely in pleuritis. The respiration is as it were rough in pneumonia, there is friction in pleuritis. Course of pneumonia is more rapid and more regular; the alternations as to better or worse are much less marked. There are intermissions and accessions of dyspnœa in pleuritis.

In chronic pneumonia and chronic pleuritis we have dulness, absence of the respiratory murmur, bronchial respiration, resonance of the cry and voice, emaciation, fever continued or intermittent; but chronic pneumonia is much more uncommon in children than chronic pleuritis, except that which accompanies pulmonary tubercles. Respiration more rarely bronchial in effusion; œgophonic resonance of the voice and cry, a sign somewhat difficult of detection; variations in the extent of the dulness and of the absence of the respiratory murmur more frequent in pleuritic patients, which is owing to the occasional variations in the quantity of the effusion.

*Infiltration of the lung* is oftentimes accompanied, in new-born infants, with dulness, extreme feebleness and sometimes absence of the respiratory murmur. Percussion is less painful in sanguineous infiltration of the lung than in inflammation of the pleura; but we can hardly reckon on this sign, because very frequently, without the existence of pleuritis at all, mere moving of the child makes it cry. The dulness does not change its seat, according as the position is changed, as in pleuritic effusion; there is neither œgophony nor pleuritic friction. The dyspnœa is less than in pleuritis; no febrile re-action, cough more rare. Instead of tossing about the patient remains immovable; the respiration is less frequently accelerated; sometimes it is even retarded. Sometimes the subcrepitous, crepitous or sibilous râles; but these are heard very close to the ear, so as to exclude the idea of an effusion, and besides they are not heard in all the stages of the disease. Debility occasioned more promptly than by pleuritis; the cry is much more frequently veiled. Pulmonary engorgement is still more difficult to be distinguished from pleuritis, when the latter exists without effusion, especially when not accompanied with dulness; but in most cases this engorgement occasions a diminution in the sonorousness, which does not occur in pleuritis without effusion; to which we may add the veiled cry, the existence of râles, feeble respiration, and the frequent absence of fever.

*Gangrene of the lung* will be distinguished from pleuritis by bronchial or cavernous respiration, the great mucous or cavernous râle, the strong resonance of the cry or voice, fetid breath, and by copious, purulent, and fetid sputa.

Like *pulmonary phthisis,* chronic pleurisy produces frequent fever, emaciation and marasmus. In these cases, hereditary circumstances, previous history of the child (frequent colds, spitting of blood), the occurrence of tubercles in other organs, may induce us to presume the existence or absence of pulmonary tubercles. Besides, in chronic pleuritis, there are signs of pleuritic effusion, and in tubercular phthisis we hear the mucous, sub-crepitous râles, in different points, more especially in the upper parts.

In *chronic bronchitis,* which might be confounded with chronic pleuritis, the signs of effusion are wanting; it is accompanied by mucous, subcrepitous, and sibilous râles.

In *acute bronchitis* the mixture of the mucous râle, of the grave ronchus and of the rude or rough respiration, might be taken for the pleuritic friction; sometimes also there are fits of dyspnœa, as in pleuritis. But the râles of bronchitis, so variable in children, are soon replaced by others less confused; there are no pains in bronchitis, and the patient can lie indifferently on either side.

In *croup* there are accessions of dyspnœa, as in pleuritis, but more constant. Croup is rare in very young children. Sometimes there is a veiled cry in pleuritis, as in croup, in children of this age only. In croup and in angina tonsillaris, there is sonorousness of the chest, notwithstanding the diminished respiratory murmur; laryngeal sibilance in inspiration, increasing difficulty of respiration.

In *acute asthma,* the lassitude and depression are greater than in pleuritis; pulse irregular, dyspnœa continued, and constantly increasing in intensity; breathing more laborious. *Chronic asthma,* a rare occurrence in children, is accompanied by symptoms of chronic bronchitis, and by those of pulmonary emphysema. No pain of side, no fever, no signs of effusion. In *pleurodynia,* the absence of all signs of effusion and of false membranes, sometimes an increase of pain on moving, no cough, fever none, or almost none. It is evident how difficult of detection pleurodynia is in very young children. Pressure or percussion of the chest might occasion pain, as in pleurisy; but then there would be absence of the signs of effusion, modifications in the intensity of the respiratory murmur, and some cough.

In *pericarditis* with effusion into the pericardium, which might be confounded with pleurisy, there is dulness in the præcordial region; pulsations of the heart dull, deep, sometimes irregular; pulse frequently irregular, sometimes a friction sound isochronous with the beats of the heart, whilst the pleuritic friction is isochronous with the respiratory movements. Besides the pleuritic friction is heard almost always posteriorly and laterally.

In certain *pleuritic changes not inflammatory,* such as sero-sanguineous or serous effusions, so frequent in new-born children without the coincidence of pleuritis; dulness of sound, dyspnœa, interrupted respiration, weak cry, great disturbance; but no febrile re-action.

In *pneumothorax*, there is gaseous exhalation into the cavity of the pleura, independent of the inflammation of this membrane, which simulates pneumothorax of an inflammatory nature. In a case observed by the author at the Hôpital des Enfans there was intense dyspnœa, pulse 172 and regular, very little disturbance; skin dry, a little hot; lips dry and purple; great dyspnœa. The patient lies on the abdomen, inclining to the right side; respirations 48, and short, with rising of the thorax. Sonorousness of the chest very great, especially on the right side, which seems a little dilated. Percussion a little painful on the right. The vesicular respiration is heard on the left without any râle. On the right there is complete absence of respiration over every region. The child died. The pleura contained air, and this membrane was perfectly healthy. No other morbid change in the chest, except grey hepatizations, partial over the right lung, and some emphysematous patches over the left.

*Prognosis.*—The prognosis of pleuritis in children is worse, the younger the patient happens to be. The chief danger of the disease is to be attributed to the debility, and to the frequency of accompanying affections. The disease is more dangerous in weak and debilitated patients, than in those of a strong constitution, and who are well formed. One condition which renders the prognosis worse in delicate children, is, that in general the coincidences of affections of other organs are more frequent in them than in robust children. Still, in the latter, the cerebral complications accompany pleuritis a little more frequently. Death comes on much sooner when these complications exist. According to the author the pleurisies of summer terminate in death much more frequently, than those of any other season of the year. Pleurisy, coming on in the course of eruptive fevers, is generally of a serious character.

*Treatment.*—The treatment of pleurisy in children is attended with considerable difficulty. Some, as Triller, Vebecker, &c. advise bleeding in the pleuritis of children. Most authors reject phlebotomy, advising it to be confined to very rare cases. Several, says the author, recommend us to substitute for general bleeding, leeches, and cupping-glasses, their mode of action having the advantage of not being immediate or sudden, in weak subjects, and also because leeches applied over the painful region act as a good derivative. In young children the small size of the vein renders general bleeding difficult, and it is usual to replace it by leeches applied over the parietes of the thorax on the affected side. In infants not yet one month old, a single leech is sufficient, two or three in children of a few months old; four or five in children of from three to four years of age. After the fifth or sixth year the local bleeding may often be replaced with advantage by general bleeding, which produces an immediate depletion more easily graduated and limited, and which irritates the child less.

*Purgatives.*—The author has met several cases of gastro-intestinal inflammation (mucous) occasioned by purgatives, and especially by tartaremetic. Hence the necessity of administering purgatives in small doses in the case of children, and of preferring, more especially in new-born children, those which are least active. The repetition of purgatives is not to be dreaded, as that of bleeding, in the treatment of children. They should, however, be administered in small doses, in which case they are observed to promote the absorption of any effusion that may exist in a remarkable manner. They should not be employed till after the inflammatory period of the disease, after the cessation of pain, and the development of the effusion, unless constipation exists at the commencement, as sometimes happens. It is in chronic pleurisy chiefly that purgatives may be found useful. Their chief efficacy depends on their revulsive powers. *Emetics* have been extolled for their efficacy in the treatment of pleurisy, more especially that of the verminous character. *Diuretics,* more especially the nitrate of potass and oxymel of squill, are strongly recommended by our author. *Blisters.*—These are generally placed over the chest on the affected side, on a level with the part affected. Some practitioners have observed, that, where blisters were allowed to suppurate in children, they soon became gangrenous. They should not be employed till the disappearance of the inflammatory symptoms. When the pleuritis is occasioned by the suppression of a cutaneous affection, the principal indication is to endeavour to bring it back by means of a blister placed over the region where the eruption was chiefly seated.

*Other Cutaneous Derivatives.*—Hot baths, sinapisms applied to different parts of the body, dry frictions, mustard pediluvia; the various species of sudorifics; frictions with croton oil; tartar-emetic to the skin, &c. &c. Burgundy-pitch and diachylon plaster with gum, which, in little patients whose skins are sensitive, keep up a slight irritation, and a mild and uniform heat. The author gives a very useful admonition with respect to the temperature of the sleeping-room of the patient, also in reference to the quantity of clothes worn by him, whilst confined to bed. He has seen, when the disease was advancing rapidly towards a cure, that the effusion became suddenly increased, in consequence of the child being allowed to leave the bed for a moment.

*Opiates.*—To calm the pain, cough and other troublesome symptoms, various preparations of opium have been found serviceable. *Tonics.*—In chronic pleurisy it is necessary to support the strength by means of tonics and nourishing food. In the case of children however great reserve is necessary. *Diet.*—In acute pleurisy, on the contrary, strict attention to diet is required; in infants at the breast even, during the inflammatory stage, the nurse's milk should be suppressed, so however as not to produce excessive debility, which is always injurious. In children old enough to support the privation of aliments, only a little milk should be allowed. In children the want of nutrition is active and pressing, and accordingly becomes a more active means of treatment than in persons more advanced in years, by affording more facility to the absorption of effusions.

### THERAPEUTICS OF CHILDREN FROM TWO TO FIFTEEN YEARS OF AGE. By A. BECQUEREL, Intern in the Hôpital des Enfans.

*The Therapeutics of Children.*

When called to treat a child, we must commence by taking into consideration several conditions connected with the physiological or pathological state of the young patient. We shall now examine these different conditions, and what modifications they render necessary in the treatment.

1st. *Physiological Conditions.*—In infancy a great organic condition predominates over all others, that resulting from the necessity of growth. The nutritive action of composition exceeds that of decomposition, because it is necessary that it should not only serve to renew the parts already existing, but because it has also to produce new molecules destined for the development and growth of the several tissues. The latter are endowed with greater vital activity than in the adult, the interstitial nutritive movement being more active, and it being in some respects double. It is to this great condition that we should refer the predominance of the nervous system in infancy and early age, and the more easy and rapid calling forth of the several sympathies, and all the consequences flowing from these two conditions. When disease occurs in a child, the first effect is a more easy production of nervous symptoms and of sympathetic phenomena, which must be taken into serious consideration. As soon as the disease becomes lengthened, and strict diet is practised, the nutritive movement of composition becomes arrested, and instead of predominating over that of decomposition, it does not even balance it; then the child becomes rapidly debilitated. This debility is produced still more easily and more rapidly, when the remedies employed are of a depleting character, such as blood-letting and purgatives. We should therefore have this fact present to our minds, as one resulting from the organization of children. The age of the child is another condition which must not be lost sight of; it is beyond a doubt that the younger they are, the more striking and prominent will be the characters just mentioned; whilst, as the child advances from twelve to seventeen, they become less marked, and admit more active treatment. Thus in a child of from twelve to fifteen attacked with pneumonia, there will be no reason to fear the effects of general bloodletting; on the contrary, such a remedial agent will be found very advantageous. The constitution of children must exercise considerable influence over the treatment employed. Thus, if we have to do with a child with a very fair, and, as it were, a transparent skin, flesh rather flaccid, fair hair, blue eyes, and long and black eye-lashes contrasting with these other conditions, a subject, in fact, presenting all the attributes of that state to which the name of lymphatic constitution is conventionally given, it is certain that in such an individual the interstitial nutritive movement is less active than it should be; wherefore diseases will pull such a patient down much more readily—in such a case evacuants must be employed with considerable reserve, and tonic remedies capable of restoring the exhausted vital powers must be resorted to as early as possible. The same thing holds good of a child placed in unfavourable and unhealthy circumstances, with respect to habitation, aliment, &c. Sex exercises some influence on treatment.

*Pathological Conditions.*—If the physiological state exercises powerful influence over the treatment of the diseases of children, the pathological state must *à fortiori* possess similar influence. Now the great pathological conditions which the physician must have present to his mind, are the following:—

1st. *The Nature of the Disease.*—Diseases, whether local or general, are far from resembling each other. Now, the treatment must vary with the nature of the diseases; thus no practitioner will take it into his head to treat inflammation of an organ or of a tissue, by the same remedial means, as he would treat hæmorrhage, or tubercular degenerescence of such organ or tissue.

2nd. *The Local State.*—The seat of the disease must be taken into consideration, and be well established by a sure diagnosis, a thing far from being always easy in the case of children. It is against it that what are called local means must be directed; no one, for instance, would think of applying leeches to the abdómen or to the mastoid processes in the treatment of pleuritis or pneumonia.

3rd. *The General State.*—In children, in consequence of their organization, the nervous system is much more easily implicated than in adults, and the sympathies are brought into play much more rapidly and intensely. The result of this is, that the general state is often the more prominent, and accordingly absorbs the attention of the physician. When this is the case, the physician, whilst taking into consideration the local state, must endeavour to combat this general hypersthenic state. In the consideration of these phenomena there are valuable indications to guide him in the application of his therapeutic agents. In some cases, the general state is every thing, and the local lesion little or nothing; it is then against the former he must act with energy, and often overlook the second.

4th. *Previous Diseases.*—When a disease becomes developed in a child during convalescence from some other, a thing far from being uncommon, the new affection finds the young patient already enfeebled, and consequently less able to resist, as well the disease itself, as the debilitating means to be employed in its treatment. The latter, therefore, must be employed with reserve; such reserve is particularly necessary in convalescence from eruptive and typhoid fevers. When the new affection occurs in a scrofulous or tubercular subject, it is then such reserve is peculiarly called for.

5th. *The Acute or Chronic State of the Disease.*—In the former case, we shall derive much more advantage from depleting and revulsive remedies, whilst in the latter, the contrary will sometimes occur, and we must rather seek to strengthen our young patient, so as to enable him to resist the debilitating influence of a lingering disease.

6th. *The Stage or Period of the Disease.*—It is indisputable that, in the case of children, the sooner we can combat a disease such as an inflammation, for instance, the more hope shall we have of getting it under; yet, if we are not called in till a much later period of the disease, we shall have to act of course, but with more energy; unfortunately, however well-directed and active our remedial measures may then be, they are too frequently unsuccessful.

7th. *The Appreciable Causes.*—When the disease appears to be developed under the influence of an external cause, as of cold, for instance, we may hope more, and adopt with more energy an active line of treatment; whilst if the cause is internal, if we have to do, for instance, with those numerous inflammations, which follow measles and scarlatina, such as bronchitis, pneumonia, ophthalmia, &c. the antiphlogistic treatment succeeds much less than when the disease arises from an external cause. The same principle holds still more strongly, when we have to do with inflammation connected with tubercles; here we may assure ourselves beforehand that antiphlogistics will not only be unavailing, but that they will even contribute to accelerate the fatal term of the disease.

8th. *The Treatment previously employed.*—Thus when we know that a pneumonia, for instance, has been already treated by blood-letting, general and local, we shall be reserved in employing these means again, and we shall then adopt blistering.

9th. *Action of Therapeutic Agents.*—It is difficult to establish any general rules on this point in the case of children. The greatest differences are observable in them, as well as in adults. Such a medicine, opium for instance, is borne very well by a child five years old, which produces in another of the same age, and apparently of the same strength, serious effects, as narcotism. If some children are able to bear large doses of certain medicines, such a thing is by no means always the case, and frequently very small quantities produce bad effects. We should, therefore, lay it down as a principle, in the therapeutics of children, always to commence with very small doses of active medicines. Such are the general principles which should guide the physician in the treatment of the diseases of children. We shall now pass in review the principal methods of medication, and study their influence on the diseases of early age.

### ANTIPHLOGISTIC MEDICATION.

*Blood-letting.*—We shall consider separately, general blood-letting, and local bleeding by means of leeches or the cupping-glass.

*General Bleeding.*—Several practitioners proscribe this mode of treatment, especially before the age of 12. It is said, and with good reason, that it debilitates children, and throws them into such an anemic state, that it, independently of the disease, causes them to sink; or that it favours the development of various secondary diseases. In many cases this is certainly true, especially when we have to deal with children who are predisposed to tubercles in consequence of previous diseases; still this is no reason for proscribing them altogether. In children of any age, especially from four or five to fifteen years, should a pneumonia or pleuritis come on, in the midst of good health, and where the constitution is good, should such inflammation be accompanied by strong febrile re-action, heat of skin, &c. considerable advantage may be derived from a bleeding at the onset of the disease; it may even be repeated if the fever is not diminished, and the disease is not relieved; should the disease continue after the second bleeding, local blood-letting is then to be preferred. Should acute bronchitis of some extent come on in children circumstanced as now mentioned, and if there be considerable embarrassment of the breathing, general blood-letting, even in young patients, generally diminishes the dyspnœa and brings down the fever. The same indication is presented in amygdalitis without false membranes, and accompanied by intense fever. The same may be said of every acute inflammation producing strong re-action. In general we should employ but a single blood-letting; by repeating the operation we run the risk of debilitating the little patient too much. However, if in several acute inflammations attended with violent pain, we often find our account in bleeding children, this is far from being always the case. Thus, if this inflammation, a thing very frequent, be connected with a more general disease of which it is but one of the elements, such as measles, scarlatina, small-pox, &c. general bleeding will be rather injurious than beneficial; inasmuch as these diseases have already a peculiar debilitating influence on the system. Though general bleeding be not always attended with success, yet in lobular pneumonia occurring even in very young children it should be employed, but with caution, in preference to leeches. When this pneumonia depends on a tubercular affection, general bleeding should be avoided. The same reserve applies to delicate subjects of a lymphatic constitution. As a general principle it may be laid down, that in children bleeding should be seldom employed in the treatment of cerebral affections. In such cases local bleeding is to be preferred. With respect to the quantity to be taken, it is difficult to lay down any rule of universal application. In a word, when we employ bleeding in the treatment of children, we should always do it with the utmost moderation, and have present to our minds the possibility of the development of an anemic state, from which they may not easily emerge, and which may engender secondary diseases more or less serious and even fatal.

*Local Blood-letting* (by leeches and the cupping glass).—Generally speaking moderate local bleeding is probably attended with less inconvenience than the preceding; but when it is copious and immoderate, it becomes still more dangerous than general bleeding, the quantity of which can always be better appreciated. In all children, but especially in the case of those who are very young, in those who have a fine, delicate skin, or a lymphatic constitution, or in the case of those who have been debilitated by unwholesome lactation, or subsequently by insufficient diet, or again by the previous existence of another disease, local or general, as eruptive or typhoid fever, the application of leeches may be attended with serious consequences.

1st. *Hæmorrhages.*—Here we have a constant oozing of blood through the leech-bites, which cannot be arrested without the utmost difficulty. Hence the application of leeches to children, or to those presenting the condition above mentioned, requires great caution. The leech-bites may become inflamed, and ulcerate; we have sometimes seen them become gangrenous. These are very unpleasant consequences. Local bleeding is useful in many diseases; in some, practitioners differ in opinion with respect to its expediency; in others it is decidedly mischievous. We shall consider these different cases. In every acute local inflammation, when freely developed, the indication of local blood-letting is positive. Thus, in pneumonia, pleuritis, bornchitis, peritonitis, enteritis, &c. this practice is indicated; the number of leeches, however, must be regulated by the age and strength of the young patient, as also by the severity of the disease; nor need we fear a repetition of the application, care, however, being taken not to exhaust the child too much. When there exists an acute pain in any point, the application of a few leeches is indicated. The same may be said in the case of acute articular rheumatism, a disease however very uncommon in young persons. When we observe rather intense functional disturbance of an organ, without our being able to connect it with any lesion of tissue in this organ, advantage may be derived from the application of a few leeches. In cerebral affections it is a common practice to apply leeches to the base of the cranium, and more especially to the mastoid processes, and certainly oftentimes with advantage. The great majority of cerebral affections in children are attributable to encephalic congestion, and the injection, which almost always coincides with convulsions, may be adduced as one of the proofs of the reality of this cause. We can easily understand the benefit to be derived in such cases from the employment of local bleeding combined with derivatives. This sanguineous congestion of the brain is manifestly the frequent cause of a certain number of cerebral affections; but in several cases, probably in the majority, it is itself but the consequence of an important organic lesion of the brain or its membranes: as for instance tubercles which may be developed in these parts, and more especially in the pia mater, as also acute or chronic inflammation which is the result of them. Here then is a permanent cause of stimulation; it is in fact under its influence that those sanguineous or serous congestions which produce convulsions, &c. are so frequently developed. In this case the local bleeding is employed to combat these symptoms, and sometimes it does arrest them; but most frequently it is ineffectual; as there is a permanent cause, an organic change, which acts as an unceasing stimulus. This is the reason why leeches are almost always so useless in that disease of children, which commonly goes by the name of cerebral fever, since it is owing to the development of an inflammation of the pia mater (meningitis) and this inflammation is itself connected with tubercular granulations of this membrane. Here it becomes very important to establish a correct diagnosis. If the previous history and the present state of the young patient incline us to admit the existence of tubercular meningitis, it will be almost useless to exhaust them by having continual recourse to local bleeding; in the contrary case, such practice will be beneficial. It may be observed here that the question as to whether a cerebral affection be tubercular or not, is often very difficult of solution. When it is plain that tubercles exist in some other part of the system, the thing is possible; but these tubercles are often so little advanced that they cannot be recognized, and so we are left in doubt. We must then rely on experience and on previous observation, which have shown us that in nine-tenths of the cases the cerebral affections of children are of a tubercular nature: it is admitted; such admission, however, rests only on analogy. From what we have now seen, it is obvious how far the practitioner must be from being able to affirm the cure of cerebral affections by the application of local bleeding; he may sometimes, but not always, calm such symptoms as delirium, convulsions, &c. &c. but not destroy the organic cause (tubercular meningitis) which has produced it. When the cerebral symptoms are the consequence of an acute disease developed in some other point besides the brain or its membranes, such as eruptive or typhoid fevers, various organic inflammations, in general advantage is derived from the application of leeches to the base of the cranium; this practice should be combined with *derivatives*.—Convulsions and other cerebral affections are sometimes the consequence of the anemic state, of exhaustion in fact. It is of the utmost importance accurately to diagnose such cases; as the taking of an additional quantity of blood must aggravate the disease. This diagnosis may almost always be established by considering the general state of the young patient, as also the previous history of his case. Eruptive fevers are often complicated with different inflammations, such as bronchitis and pneumonia, which are the most frequent; enteritis, ophthalmia, &c. may also be observed. Are local bleedings then beneficial? The general opinion is in favour of them. Our author's experience will not warrant him in acceding to such an opinion. Such inflammations, he considers, receive from the disease which has given rise to them a peculiar and specific character, which renders them different from what they would have been, had they been openly developed, and causes them to resist much more obstinately local bleedings, which have then no other effect save that of exhausting the young patient without accomplishing a resolution of the disease. So that in such inflammations we should be very reserved in employing blood-letting, so as not to repeat it too often, and not to prescribe it, unless there be intense functional disturbance of the diseased organ; such, for instance, as a distressing dyspnœa. Those ophthalmias which so frequently complicate eruptive fevers, very often resist leeches; local active applications are then required, such as the nitrate of silver and powerful revulsives. In the typhoid fevers of children, especially when the patients are of a good constitution, leeches are to be employed only when severe abdominal symptoms exist, or some affection of the brain; caution, however, is necessary in all such cases. The development of tubercles in the different organs of the body oftentimes occasions inflammation at some certain period of their existence. We have already noticed this when speaking of tubercular meningitis; but we sometimes observe tubercular pneumonia, pleuritis and peritonitis of the same kind—in such cases local bleeding is ordinarily employed. It cannot be denied that this means calms the symptoms, diminishes the functional disturbance, and the fever; but we must not deceive ourselves with respect to the amendment which takes place; for if it is sometimes definite, it is not always so, and the young patient is often destined to perish at a later period, either by the ulterior progress of the tubercles, or by the development of a new inflammation around them. In these cases, however, we must employ leeches, but with moderation, for the purpose of diminishing the febrile re-action, and so of enabling us to employ other plans of treatment. It is almost useless to endeavour to combat tubercular enteritis by local bleeding. However we sometimes, though rarely, find tubercular ulcerations cicatrised, or in the way of being so. In such cases it is nature and not art that effects the cure. The cupping-glasses do not present the same inconveniences with respect to hæmorrhages, ulcerations, &c.; their application is also more easy and less painful. They cannot, however, be employed in young patients when very much emaciated. They are prescribed in the same cases as leeches, for diseases of the chest and abdomen. We must necessarily have recourse to the latter, when we would practise local bleeding at the base of the cranium.

